# Nationwide Assessment of Water Quality in Rivers across Lebanon by Quantifying Fecal Indicators Densities and Profiling Antibiotic Resistance of *Escherichia coli*

**DOI:** 10.3390/antibiotics10070883

**Published:** 2021-07-20

**Authors:** Lea A. Dagher, Jouman Hassan, Samer Kharroubi, Hadi Jaafar, Issmat I. Kassem

**Affiliations:** 1 Department of Nutrition and Food Sciences, Faculty of Agricultural and Food Sciences, American University of Beirut (AUB), Beirut 1107 2020, Lebanon; lad17@mail.aub.edu (L.A.D.); sk157@aub.edu.lb (S.K.); 2 Center for Food Safety, Department of Food Science and Technology, University of Georgia, 1109 Experiment Street, Griffin, GA 30223, USA; Jouman.Hassan@uga.edu; 3 Department of Agriculture, Faculty of Agricultural and Food Sciences, American University of Beirut (AUB), Beirut 1107 2020, Lebanon; hj01@aub.edu.lb

**Keywords:** water quality, rivers, fecal pollution, fecal indicators, fecal coliforms, *E. coli*, antibiotic resistance, agriculture, recreation, Lebanon

## Abstract

The use of contaminated water has been associated with severe disease outbreaks. Due to widespread pollution with untreated sewage, concerns have been raised over water quality in Lebanon, a country with well-documented challenges in infrastructure. Here, we evaluated the water quality of major rivers in Lebanon by quantifying the densities of fecal indicator bacteria (fecal coliforms and *Escherichia coli*). Additionally, we assessed the dissemination of antibiotic-resistant *E. coli* in river water. Composite water samples (*n* = 132) were collected from fourteen rivers, and 378 *E. coli* were isolated and analyzed. Fecal coliforms and *E. coli* were detected in 96.29% and 95.5% of the samples, respectively. Additionally, 73.48–61.3% and 31.81% of the samples exceeded the microbiological acceptability standards for irrigation and the fecal coliform limit for recreational activities, respectively. The *E. coli* exhibited resistance to ampicillin (40% of isolates), amoxicillin + clavulanic acid (42%), cefepime (4%), cefotaxime (14%), cefalexin (46%), cefixime (17%), doripenem (0.3%), imipenem (0.5%), gentamicin (6%), kanamycin (9%), streptomycin (35%), tetracycline (35%), ciprofloxacin (10%), norfloxacin (7%), trimethoprim-sulfamethoxazole (32%), and chloramphenicol (13%). Notably, 45.8% of the isolates were classified as multidrug resistant (MDR). Our results highlight the need to urgently address fecal pollution and the dissemination of antibiotic resistance in Lebanese rivers.

## 1. Introduction

Natural water resources such as rivers are vital assets with a substantial impact on human health, food production, and the economy. The increase in the human population has intensified demand on water resources for both critical needs, such as agriculture and sanitation, and recreation. Furthermore, threats like pollution and climate change have contributed to water scarcity and the deterioration of water quality, increasing further the pressure on vital water resources and their sustainability worldwide [1,2,3]. 

The association of water pollution with adverse impacts on human health and the contamination of food is well documented [4]. While around 62% of irrigated lands worldwide primarily rely on surface waters [5], contaminated waters have resulted in outbreaks of disease with considerable incidences of morbidity and mortality, especially in children and other vulnerable populations [2,6,7,8]. This is not surprising because polluted water is known to harbor a variety of microbial pathogens, including bacteria, viruses and parasites, and chemical contaminants. For example, in the United States of America (US) recently, exposure to contaminated recreational water has resulted in outbreaks caused by *Shigella* (California), norovirus (Maine), or Shiga toxin–producing *Escherichia coli* (Minnesota) [9,10]. Furthermore, irrigation water has been potentially linked to the contamination of leafy greens, which was associated with several foodborne disease outbreaks in the US caused by *E. coli* O157:H7 and *E. coli* O145 [11]. Therefore, pollution is a serious unfolding problem that threatens water quality and requires immediate attention. 

An emerging and significant risk associated with water pollution is the emergence and/or dissemination of antibiotic-resistant (ABR) bacterial pathogens that can cause life-threatening and difficult-to-treat infections [12,13]. In fact, the World Health Organization (WHO) recognizes antimicrobial resistance as one of the most urgent public health threats that is associated with widespread suffering and economic losses. Similarly, the United Nations Environment Programme (UNEP) has designated antimicrobial resistance as one of the top six emerging environmental issues [14]. Surface waters are readily contaminated with antibiotics and resistant bacteria from a variety of sources and activities [13,15,16] such as the direct disposal of untreated wastewater into water resources, including rivers, or via runoff from agricultural fields amended with manure [17,18,19]. Wastewater is considered a reservoir of ABR bacteria and can also contain excreted and/or discarded antibiotics used for medical and/or agricultural purposes [20]. Notably, 30–90% of some antibiotics can be excreted unmetabolized from humans and animals after consumption [21,22]. These antibiotics will then exert a pressure on bacterial communities driving the selection, evolution, emergence, and/or persistence of antibiotic-resistant bacteria, which are then disseminated by water to humans and animals [21,22]. 

Water pollution and associated problems, including the spread of ABR, are exacerbated in developing countries due to several factors that include debilitated infrastructure, the lack of proper sewage and waste disposal systems, and weak water quality surveillance programs [23]. Notably, poor water quality and sanitation have been linked to 80% of all diseases, while ~1.8 million people die yearly due to waterborne diseases in developing countries [24]. The latter has an indelible impact on fragile socioeconomic development, increasing the cycle of poverty and suffering in these countries [24,25]. The World Bank estimates that water pollution can claim approximately a third of economic growth in some countries [26]. Consequently, there is a paramount need to monitor water quality and devise interventions and recommendations to limit the multi-pronged impacts associated with the pollution of water resources, especially in developing countries with substantial deficiencies in resources and infrastructure. 

Lebanon is a developing Mediterranean country with numerous challenges that include a weak infrastructure, severe economic crisis, political unrest, and widespread pollution among others. In comparison to other countries in the Middle East and North Africa (MENA) region, Lebanon is considered to be relatively water rich [19,27,28]. However, water in Lebanon have been suffering from chronic mismanagement, partially due to the absence of a national policy for integrated water resources management, which prevents the country from exploiting this valuable resource [28]. Furthermore, water in Lebanon has been under an increasing pollution threat, mainly due to (1) population growth, including an influx of ~1.5 million refugees (~1 refugee per 4 nationals) since 2011, (2) wastewater and solid waste mismanagement, and (3) absence of monitoring and surveillance programs [29,30,31,32,33]. Notably, in 2016, it was reported that only 58.54% of buildings in Lebanon were connected to a sewer network, while the rest (41.46%) use cesspools, septic tanks, or directly dispose untreated sewage into aquatic environments such as rivers and streams. It was also estimated that only 11.65% and 6.87% of the population in the North of Lebanon and Beirut (capital of Lebanon) and Mount Lebanon were connected to serviceable sewage networks, respectively [33,34]. Additionally, sewage water is not properly treated, because there is insufficient number of wastewater treatment plants (WWTPs) in Lebanon, while available WWTPs provide preliminary treatment, operate with limited capacity and budget, or lack a sewage network, rendering the plants largely nonoperational [33,34,35]. As a result, 92% of the collected wastewater are disposed of without any prior treatment into aquatic environments [33]. The aforementioned pollution strongly suggests that surface water might be widely contaminated and constitutes a reservoir that disseminates contaminants such as antibiotic resistant pathogens to other vital resources, including the food chain. Recently, multiple reports have highlighted a rise in antibiotic resistance in Lebanon [30,36,37,38,39,40,41,42] due to the abuse and misuse of antibiotics in humans and agriculture [43]. This problem appears to be widespread, with multidrug and extensively drug-resistant bacteria detected in clinical settings [43,44,45], farmed animals [36,38,46,47,48], and the environment [30,37,39,40]. However, studies on the occurrence of ABR in polluted surface waters, especially rivers, are sparse and limited in Lebanon [19,27,49].

Lebanon depends on water for agriculture (60% of water withdrawal) and municipal (29%) and industrial use (11%) [50]. Furthermore, it was estimated that 45% of the irrigated lands in Lebanon rely on surface water as a primary source [3]. Consequently, water pollution in Lebanon poses a significant risk to public health and the economy. Here, we assessed the water quality and occurrence of antibiotic-resistant bacteria in all major rivers (*n* = 14) across Lebanon. For this purpose, we quantified indicators of fecal pollution, fecal coliforms, and *E. coli* [51,52,53], from samples collected from upstream, midstream, and downstream of each river. This is important because high densities of fecal indicators have been associated with the occurrence of pathogenic microorganisms such as *Salmonella* and *E. coli* O157:H7 that have serious impact on human health [51]. Furthermore, antibiotic resistance was evaluated using *E. coli* isolated from the water because this bacterium has also been used as an indicator for monitoring the emergence and proliferation of resistance in bacterial communities [54,55,56]. To our knowledge, this is the first nationwide study that assessed water quality and antibiotic resistance across all rivers in Lebanon.

## 2. Materials and Methods

### 2.1. Collection of Water Samples from Rivers across Lebanon

Freshwater samples were collected from 14 major perennial rivers across Lebanon (May–July 2019). Two of these rivers, the Assi and Hasbani, are transboundary. Each river was divided into three sampling sites, upstream (U), midstream (M), and downstream (D), that were ~7–42 km apart depending on the length of the river and accessibility of the location. For the Litani river, which is the longest (>165 km) and largest river in Lebanon, three midstream (M) locations were included in the sampling. Composite samples were aseptically collected in triplicates from each sampling site by submerging a sterile 1 Liter Nalgene^®^ water bottle, 20–30 cm underwater without disrupting the sediment as recommended by the US Environmental Protection Agency (EPA) [40,57]. A total of one-hundred and thirty-two (*n* = 132) freshwater samples from 44 locations (Table 1) were transported to the laboratory in coolers (2–5 °C) and processed within 12–16 h of collection.

### 2.2. Quantification of Fecal Coliforms and E. coli Densities

To determine the number of colony forming units (CFU) of fecal coliforms and *E. coli*, the water samples were filtered (100 mL and 500 mL) through a 0.22-µm Millipore^®^ membranes (Sigma-Aldrich, St. Louis, MO, USA). The membranes were transferred onto RAPID’*E. coli* 2 agar plates (BioRad, Hercules, CA, USA) that were incubated at 44 °C for 18–24 h under aerobic conditions [37,58]. Typical CFUs of fecal coliforms (blue) and *E. coli* (violet to pink) colonies were counted and reported as CFU/100 mL water. The microbiological quality of the samples was determined by comparing the fecal indicator loads to the United States Environmental Protection Agency (US-EPA) standards for recreational water (permissible limit of fecal coliforms; 800 CFU/100 mL) [51] and the SEQ-EAU-2003 standard for irrigation (permissible limit of thermo-tolerant coliforms; 100 CFU/100 mL) [59].

To facilitate comparison between fecal coliforms and *E. coli* counts, bacterial densities were averaged from the triplicates of each sampling location, and the data were reported as average counts (CFU/100 mL) with standard error. The student t-test was then used to compare the average counts of *E. coli* and fecal coliforms at each location. A *p*-value < 0.05 was used to identify statistically significant differences.

### 2.3. Assessment of the Antibiotic Resistance Phenotypes of the E. coli Isolates 

Antibiotic resistance profiles of the *E. coli* isolated from water were determined using the disk diffusion assay [60]. A total of 378 *E. coli* isolates (3 colonies per sample) were purified. Random colonies (*n* = 60) were selected and their identity further confirmed using species-specific PCR analysis as described elsewhere [30,37]. All the *E. coli* (*n* = 378) were suspended in cation-adjusted Muller–Hinton (MH) broth (Oxiod, Hampshire, UK) and the turbidity was adjusted using a 0.5 McFarland standard and a spectrophotometer (Thermo Fisher Scientific, Waltham, MA, USA) [39,58]. The bacterial suspensions (100 µL) were spread onto MH agar plates (Oxiod, Hampshire, UK) and commercially available antibiotic discs were added to the plates, which were then incubated at 37 °C for 18–24 h. The tested antibiotic discs (*n* = 17) belonged to 9 different antibiotics classes, including 1, penicillins: ampicillin (AMP; 10 µg), 2, beta-lactamase inhibitor combinations: amoxicillin + clavulanic acid (AMC; 20 µg/10 µg), 3, cephalosporins: cefixime (CFM; 5 µg), cephalexin (LEX; 30 µg), cefotaxime (CTX; 30 µg), and cefepime (FEP; 30 µg); 4, carbapenems: doripenem (DOR; 10 µg), meropenem (MEM; 10 µg), and imipenem (IPM; 10 µg); 5, aminoglycosides: gentamicin (GEN; 10 µg), kanamycin (KAN; 30 µg), and streptomycin (STR; 10 µg); 6, tetracyclines: tetracycline (TET; 30 µg); 7, quinolones and fluoroquinolones: ciprofloxacin (CIP; 5 µg) and norfloxacin (NOR; 10 µg); 8, sulphonamides: trimethoprim/sulfamethoxazole (SXT; 25 µg), and 9, phenicols: chloramphenicol (CHL 30 µg). Penicillin (PEN; 6 µg) and erythromycin (ERY; 15 µg) were used as controls, because *E. coli* is intrinsically resistant to these antibiotics [61]. Additionally, *E. coli* DH5α was also included as a control across the experiments. Antibiotic resistance (ABR) was determined by measuring the diameter of the zone of inhibition around each antibiotic disc and comparing it with the Clinical and Laboratory Standards Institute (CLSI) [60] and the European Committee on Antimicrobial Susceptibility Testing (EUCAST) standards [62]. Antibiotic resistance profiles were analyzed using hierarchical clustering (HLC). For this purpose, the resistance or susceptibility of each isolate were coded in Excel^®^ (Microsoft, Redmond, WA, USA) as follows: −1 (resistant), 0 (intermediate), and 1 (susceptible); with the *E. coli* isolates represented in rows and the antibiotics in columns. Then the data were exported to MeV v4.6.2 software (http://www.tm4.org/, accessed on 10 June 2021) to perform HLC analysis using the Pearson correlation as a distance metric and the complete linkage method [58,63]. A graphical presentation (heat map) was generated with the upper limit (1; sensitive), midpoint (0; intermediate), and lowest limit (−1, resistant) colored green, black, and red, respectively [63].

## 3. Results

### 3.1. Densities of Fecal Coliforms and E. coli in River Water Samples

**Figure 1:**
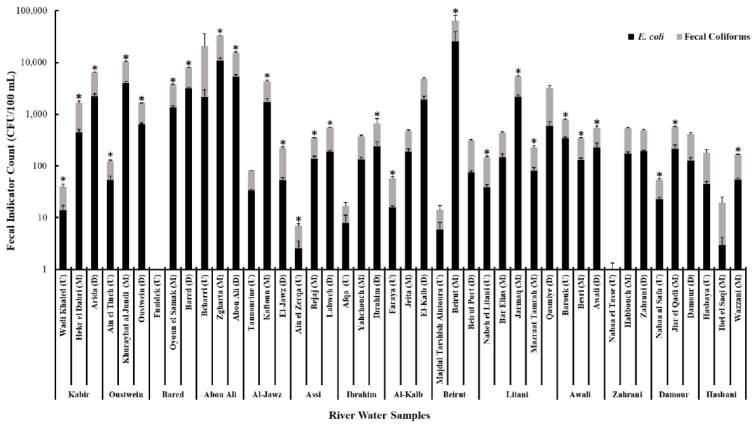
The average loads of fecal coliforms and *E. coli* counts (CFU/100 mL) in Lebanese river water. The asterisk (*) represents a statistically significant difference between fecal coliforms and *E. coli* counts (*p* < 0.05). The letters next to the sampling sites represents the location where the sample was collected, U = upstream, M = midstream, and D = downstream. Standard error bars are included with the averages.

Fecal coliforms were detected in 127 (96.2%) of 132 water samples and 43 (98%) of 44 locations (only in one location, Fnaidek, all 3 samples did not yield fecal coliforms CFUs) (Figure 1). The average number of fecal coliforms in positive locations ranged from 1 × 10^0^ CFU/100 mL to 3.66 × 10^4^ CFU/100 mL. *E. coli* was detected in 126 samples (95.5%) and in 42 (95.5%) of 44 locations (Figure 1). The average number of *E. coli* in positive locations ranged from 2.6 × 10^0^ CFU/100 mL to 2.61 × 10^4^ CFU/100 mL (Figure 1). Average numbers of fecal coliforms were higher than *E. coli* in all positive locations; however, statistically higher average numbers of fecal coliforms (*p* < 0.05) were noted for 27 locations (Figure 1). The highest average counts were recorded in samples retrieved from the midstream of Beirut river (3.66 × 10^4^ CFU/100 mL fecal coliforms and 2.61 × 10^4^ CFU/100 mL *E. coli*) followed by midstream and downstream of the Abou Ali river [Zgharta (2.16 × 10^4^ CFU/100 mL, 1.1 × 10^4^ CFU/100 mL) and Abou Ali (9.97 × 10^3^ CFU/100 mL, 5.43 × 10^3^ CFU/100 mL)] (Figure 1). With the exception of Abou Ali, Awali, and Hasbani rivers, the fecal coliforms and *E. coli* counts were generally lower upstream in comparison with those from midstream and downstream locations in the majority of the rivers (Figure 1).

### 3.2. Comparison of Fecal Coliforms and E. coli Counts to Irrigation and Recreation Standards

**Figure 2:**
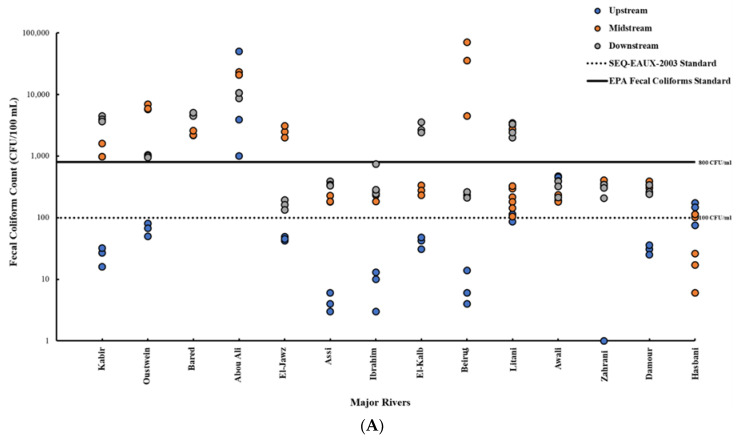

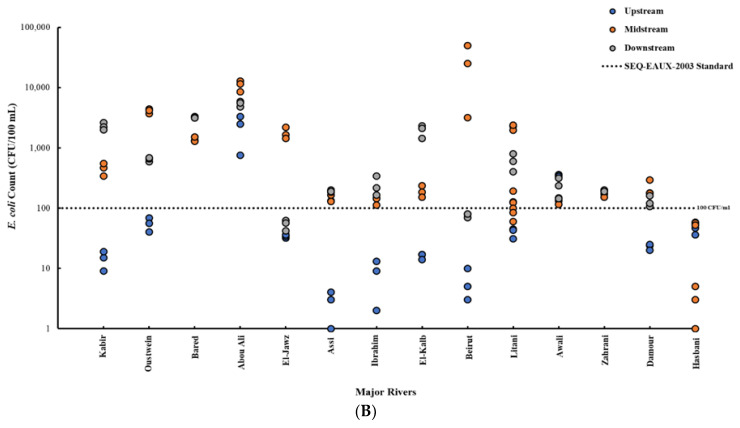
Distribution of counts of fecal coliforms (**A**) and *E. coli* (**B**) in samples collected from each location across the rivers: Upstream (blue circle), Midstream (orange), and Downstream (grey). The dotted black line indicates acceptable limit of thermo-tolerant coliforms based on the SEQ-EAUX-2003 standard for irrigation water (100 CFU/100 mL). The black line indicates the permissible limit of fecal coliforms for safe recreational water (800 CFU/100 mL) as per the EPA standards. Samples that did not yield fecal coliforms or *E. coli* are not represented in the figures.

**Figure 3:**
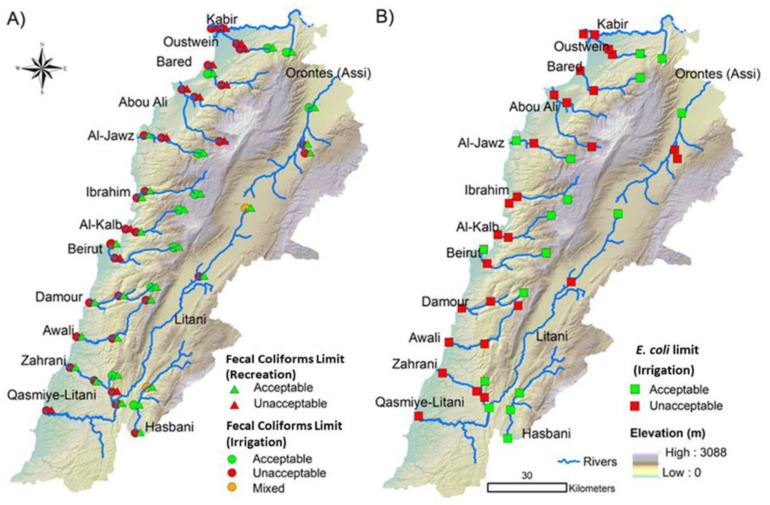
Map showing the distribution of the water samples that exceeded the acceptable limits for irrigation and recreational use. (**A**) Assessment of the acceptability of water for irrigation and recreation using fecal coliforms numbers. Red circle: all samples exceeded acceptability limits for irrigation (unacceptable); green circle: all samples were below the acceptability limit for irrigation (acceptable); orange circles: 2 samples of 3 exceeded limits for irrigation (mixed). Red triangle: all samples exceeded limits for recreation; green triangle: all samples were below the acceptability limit for recreation. (**B**) Assessment of acceptability of water for irrigation using *E. coli* numbers. Red square: all samples exceeded limits for irrigation (unacceptable); green square: all samples were below the acceptability limit for irrigation (acceptable).

Fecal coliforms and *E. coli* counts were compared with the SEQ-EAU standard (100 CFU/100 mL) for irrigation water quality. Based on fecal coliforms counts, 97 (73.48%) of the 132 water samples and 33 (75%) of the 44 locations exceeded the SEQ-EAU-2003 standard (Figure 2 and Figure 3), indicating that the water was unacceptable for irrigation. Similarly, when evaluating *E. coli* counts, it was found that 81 (61.3%) of the 132 samples and 27 (61.3%) of the 44 sampling locations exceeded the SEQ-EAU standard (Figure 2 and Figure 3). In general, most of the samples that exceeded the permissible limit for irrigation (using fecal coliforms and/or *E. coli* counts) were collected from midstream and downstream locations across the major rivers. The fecal coliform counts in upstream samples from Litani and Hasbani (2 of 3 samples/location) rivers exceeded the standard for irrigation (Figure 2). However, when considering *E. coli* counts, only upstream samples from Abou Ali and Awali rivers were found to be unacceptable (Figure 2).

The acceptability of river water for recreation was evaluated using the US-EPA standard (800 fecal coliforms CFU/100 mL). Subsequently, 42 (31.8%) of the 132 samples and 14 (31.8%) of the 44 locations exceeded the recommended standard for safe recreational use. Again, samples that exceeded the standard were collected from midstream and downstream locations; with the Abou Ali river being an exception, where all samples and locations exceeded the standard. Notably, the majority of unacceptable water samples were collected from rivers in the North (30 of 42; 71.4%) in comparison with 18% of the samples collected in the South (6 of 33) and 9% in Mount Lebanon (3 of 33) (Figure 2 and Figure 3).

### 3.3. The Antibiotic Resistance Profiles of E. coli Isolated from Water

**Figure 4:**
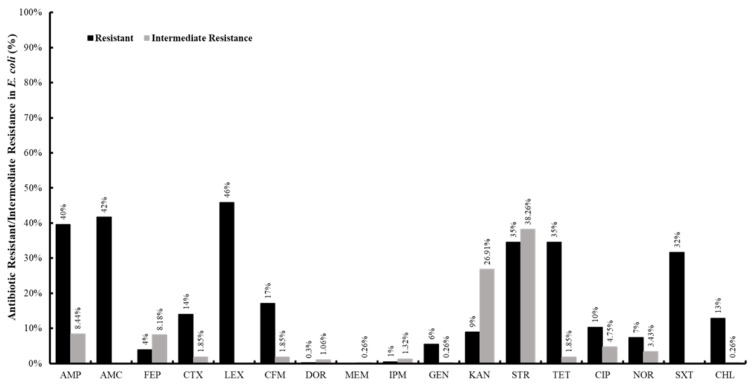
Antibiotic resistance of *E. coli* (percentage) isolated from the rivers in Lebanon. Ampicillin (AMP), amoxicillin + clavulanic acid (AMC), cefepime (FEP), cefotaxime (CTX), cephalexin (LEX), cefixime (CFM), doripenem (DOR), meropenem (MEM), imipenem (IPM), gentamicin (GEN), kanamycin (KAN), streptomycin (STR), tetracycline (TET), ciprofloxacin (CIP), norfloxacin (NOR), trimethoprim + sulfamethoxazole (SXT), and chloramphenicol (CHL). The antibiotics are arranged according to the order of antibiotics/classes listed in the CLSI guidelines.

**Figure 5:**
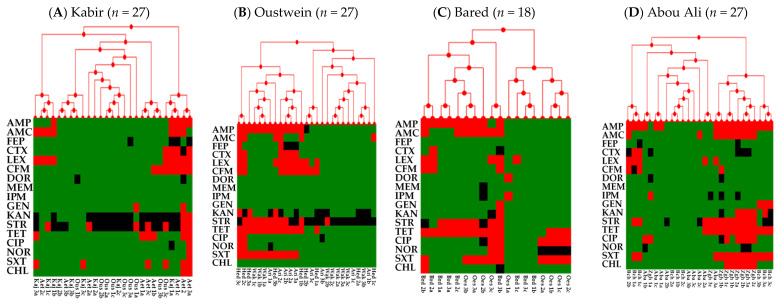

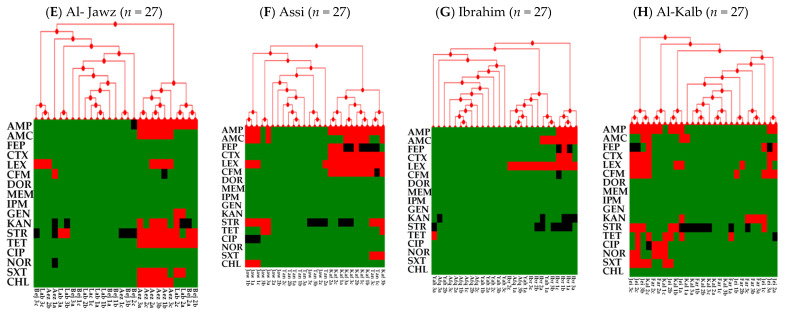

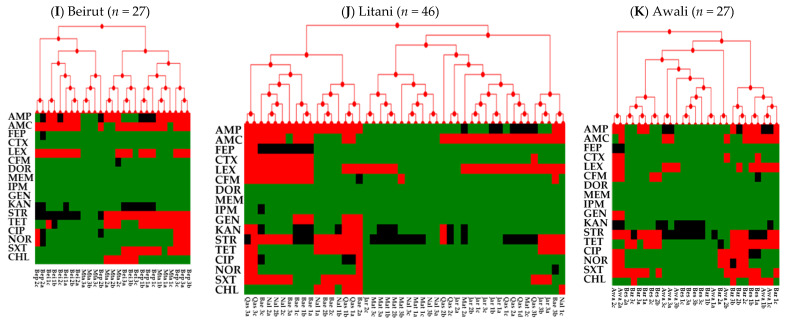

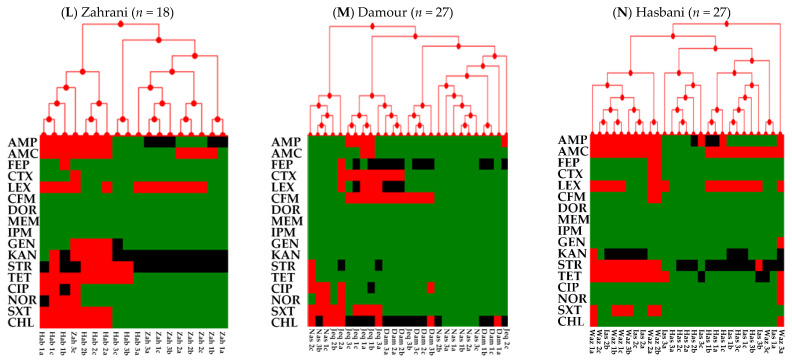
Hierarchical clustering of the antibiotic resistance (ABR) profiles of *E. coli* (*n* = 378) isolated from rivers in Lebanon. The strains were analyzed per river; n indicates the number of isolates per river. The isolates are listed on the bottom of each dendrogram. Ampicillin (AMP), amoxicillin + clavulanic acid (AMC), cefepime (FEP), cefotaxime (CTX), cephalexin (LEX), cefixime (CFM), doripenem (DOR), meropenem (MEM), imipenem (IPM), gentamicin (GEN), kanamycin (KAN), streptomycin (STR), tetracycline (TET), ciprofloxacin (CIP), norfloxacin (NOR), trimethoprim + sulfamethoxazole (SXT), and chloramphenicol (CHL). The red color in the heat map represents resistance, while black and green indicate intermediate resistance and susceptibility, respectively.

Antibiotic resistance profiles of 378 *E. coli* (3 colonies per sample) were determined. The isolates exhibited resistance to ampicillin (40%), amoxicillin + clavulanic acid (42%), cefepime (4%), cefotaxime (14%), cephalexin (46%), cefixime (17%), doripenem (0.3%), imipenem (0.5%), gentamicin (6%), kanamycin (9%), streptomycin (35%), tetracycline (35%), ciprofloxacin (10%), norfloxacin (7%), trimethoprim + sulfamethoxazole (32%) and chloramphenicol (13%) (Figure 4). All isolates were sensitive to meropenem. Furthermore, intermediate resistance was observed against several antibiotics, including streptomycin (38.26%), kanamycin (26.9%), ampicillin (8.44%), cefepime (8.1%), ciprofloxacin (4.75%), norfloxacin (3.4%), tetracycline (1.85%), cefotaxime (1.85%), cefixime (1.85%), imipenem (1.3%), doripenem (1%), meropenem (0.26%), gentamicin (0.26%), and chloramphenicol (0.26%) (Figure 4). Notably, some *E. coli* (*n* = 3) isolated from Oyoun el Samak (midstream of Bared river) and Zgharta (midstream of Abou Ali river) in the North were resistant to carbapenems (doripenem and/or imipenem) (Figure 5). Furthermore, 45.8% (*n* = 173) of the isolates were classified as multidrug resistant (MDR; resistance to at least three classes of antibiotics). Further analysis showed that 77%, 72%, 62%, 55.5%, and 47% of the *E. coli* from Beirut, Bared, Awali, Abou Ali, and Litani rivers were MDR, respectively (Figure 5). Additionally, 8.7% (33 isolates), 7.1% (27), 6.8% (26), and 0.52% (2) of the isolates were resistant to 5, 6, 7, and 8 antibiotic classes, respectively. HLC analysis of the ABR profiles of isolates from each river showed widespread resistance to AMP, AMC, LEX, CFM, STR, TET, and SXT in most of the rivers (Figure 5). Furthermore, resistance to CIP was notable in isolates from Bared, Abou Ali, and El Jawz rivers (Figure 5).

## 4. Discussion

Clean water is an integral component in the production of safe food and in maintaining human health. The use of contaminated water results in a variety of waterborne diseases and aggravates infectious diseases and the burden of foodborne illnesses, especially in vulnerable and disenfranchised populations [64,65]. Therefore, it is paramount to monitor the quality of water in order to devise mechanisms and policies that prevent the contamination of vital water resources such as rivers. Despite the critical role of rivers in sustainable agriculture and socioeconomic growth in Lebanon [5,19,27], river water quality has been confronted with a plethora of challenges, including severe deficiencies in infrastructure, wastewater management, and antimicrobial stewardship. Although it is widely known that aquatic environments are severely affected by untreated sewage and other agricultural and industrial contaminants [66,67], studies on fecal pollution and microbial safety of surface water in Lebanon are scant. For this purpose, we conducted this study to evaluate water quality by assessing indicators of fecal pollution (fecal coliforms and *E. coli*) [51] and antibiotic resistance (*E. coli*) [55,56] across all major rivers in Lebanon. 

Our data showed that 96.2%, and 95.5% of the river water samples in Lebanon harbored fecal coliforms and *E. coli*, respectively (Figure 1 and Figure 2). The widespread detection of the fecal indicators was not surprising, given that a previous report indicated that 92% of the collected wastewater in Lebanon were discarded, without any treatment, into aquatic environments, while a considerable number of buildings lacked connection to a sewer network [33,34,68]. The high bacterial loads reported in some locations such as midstream of Beirut (Beirut) and Oustwein (Khuraybat el Jundi) rivers, downstream of Bared (Bared) and Kabir (Arida) rivers, and across Abou Ali river were expected because these rivers are heavily impacted by human sewage and other urban contaminants. A report in 2016 indicated that WWTPs were either absent or operated at limited capacity to treat wastewater in the North and in Beirut, which resulted in the release of untreated wastewater to aquatic environments [33,34]. Additionally, these rivers are located in areas with high population densities, including crowded refugee camps that lack infrastructure [30,31,32,33,39]. Therefore, these rivers are affected by urban activities, highlighting the negative impact of crowding and the debilitated infrastructure on water quality. Although the fecal indicators were widely detected in river water samples, it was noted that samples collected from upstream harbored relatively lower numbers of fecal coliforms and *E. coli* as compared with midstream and downstream samples in 11 of the 14 rivers; Abou Ali, Awali, and Hasbani rivers were the exception (Figure 1). This result suggested that the river sources were likely less affected by pollution, potentially due to limited urbanization in those locations. Therefore, as expected, the pollution (densities of fecal indicators) appears to increase as the rivers cross locations with more dense populations and increasing agricultural and industrial activities.

For assessing water quality in Lebanon, previous studies relied on international standards of fecal indicators in irrigation and recreational water. Specifically, the French SEQ-EAU-2003 [59] and the US EPA standard [51,69] have been considered for evaluating irrigation and recreational water quality, respectively. According to SEQ-EAU-2003, the acceptable limit of thermo-tolerant fecal coliforms (fecal coliforms or *E. coli*) is 100 CFU/100 mL, which is similar to standards set by other countries in the European Union [70], including Spain (Royal Decree 1620/2007, December 2007) [71,72]. Consequently, we adopted these standards to assess the suitability of river water for irrigation and recreation in Lebanon. When considering both fecal coliforms and *E. coli* numbers, it was noted that 61.3–73.48% of samples and 61.3–75% of the locations exceeded the limit set by SEQ-EAU-2003 for irrigation water (Figure 2 and Figure 3). Therefore, *E. coli* densities revealed a lower number of unacceptable irrigation water samples and locations in comparison with fecal coliforms (Figure 2 and Figure 3). However, even when considering the more conservative indicator (*E. coli*), we found that a majority of the unacceptable samples were located in regions where agricultural practices are relatively concentrated, which includes the North of Lebanon (70.4%) and Beqaa (60%). Furthermore, in the South, samples from Jarmaq and Qasmiye rivers, which represent the midstream and downstream of the Litani river, exceeded the SEQ-EAU-2003 standard for irrigation. This can be attributed to the fact that the Litani river flows from the Beqaa Valley and carries sewage from different cities such as Baalbeck, Bar Elias, Zahle, Joub Jannine, and Sifri as well as effluents from informal refugee settlements and many industries (such as food factories and sugar mills), poultry farms, and slaughterhouses located in the Litani basin (Figure 3) [73]. Notably, the Litani River is the chief source of irrigation for agricultural lands in the Beqaa Valley and the South, and it has been well established that the river is being subjected to different pollutants, including pesticides and human and animal waste [73,74]. For example, ~69 villages and cities release approximately 47 Mm^3^ per year of raw sewage into the Litani River [75]. Taken together, it appears that a high number of water samples from agriculturally important rivers in Lebanon were fecally contaminated and were deemed unacceptable for irrigation. This can be further deduced by comparing the numbers with those from counties with better infrastructure and water management. For example, in Canada, of 501 irrigation water samples analyzed, only 0.8–22% exceeded the Canadian permissible limit for *E. coli* [76]. Furthermore, our findings suggest that human activities near the rivers significantly affect the safety of water, because most upstream sites, located in remote and less populated areas, were found to be suitable for irrigation (Figure 3). Regardless, the quality of river water is a serious concern, because fecally-contaminated irrigation water will affect the safety of produce, which will increase the risk of contracting foodborne infections that can cause serious or life-threatening diseases in humans [77,78]. Fecal pathogens like *Salmonella* spp., *E. coli* O157:H7, *Cryptosporidium*, Norovirus, Hepatitis A Virus among others have been associated with the contamination of produce, resulting in considerable outbreaks and/or illnesses [79]. Indeed, three studies reported that the produce such as spinach, parsley, cabbage and lettuce collected from the Beqaa Valley were contaminated with fecal bacteria [27,80,81]. Notably, fresh produce is usually consumed raw, which increases the risk of foodborne diseases. Therefore, it is of paramount importance to monitor the quality of water used for irrigation in order to control the proliferation of disease in Lebanon, which is particularly vulnerable to these infections due to ongoing severe medical and economic crises [82]. It should be highlighted that our study did not assess other types of contamination like pesticides and other xenobiotics, which perhaps further emphasizes the potential impact and scope of water pollution.

To assess the suitability of water for recreational use, fecal coliforms counts from the water samples were compared with the US EPA standard (Figure 2 and Figure 3). The data showed that 31.8% of the samples were deemed unacceptable for recreational use. Notably, the majority of the samples collected from the North (71.4%) of Lebanon were unacceptable for recreation, which is likely related to the pollution factors that were mentioned earlier. Additionally, the North has arguably more severe poverty and infrastructure challenges in comparison with the rest of the country [82]. Although 68.2% of the river samples were found to be suitable for recreational use (based on fecal coliforms counts), these results should be interpreted with caution, because (1) we only assessed fecal pollution but not other types of contamination such as chemical contaminants, (2) our sampling was cross-sectional and did not account for temporal variations in the densities of fecal indicators, (3) the sampling was done after a relatively wet season, and (4) some chemical contamination might have affected the densities of the fecal indicators. For example, pollution downstream of Beirut River (Beirut Port) from industrial, animal, and hospital wastes is well established; however, densities of fecal coliforms at this location did not exceed the EPA or SEQ-EAU-2003 standards for recreational use or irrigation. It is possible that the release of toxic chemicals (agrochemicals, detergents, chlorinated compound, etc.) might have altered the numbers of fecal coliforms in these samples [83].

It is known that even the discharge of treated sewage can release antibiotic-resistant bacteria, transmissible genetic elements that encode resistance, and antibiotics residue into environments [84,85]. Therefore, the emergence and dissemination of ABR has been linked to fecal pollution. Given that untreated sewage and other contaminants are released into Lebanese rivers and that ABR is widespread in other vital matrices in Lebanon [30,36,37,38,39,40,41,42], it was necessary to address ABR in our samples. The latter was addressed by assessing resistance of river water *E. coli*, which is normally used as an indicator of ABR [86,87]. Our data showed that 173 (~45.8%) *E. coli* were multidrug resistant, exhibiting resistance to at least three antibiotic classes (Figure 5). The percentage of multidrug-resistant *E. coli* in Lebanese rivers is slightly lower than those previously reported for sewage contaminated rivers in Romania (60.34%) [88] and in Ethiopia (78%) [89]. However, Lebanon is a much smaller country, both in size (~10,450 Km^2^) and human population (~6.8 million) and has comparatively limited agricultural and industrial output, which perhaps reveals the severity of ABR prevalence in Lebanese river water. The latter can be further evaluated, when considering countries with better wastewater management systems. For example, MDR *E.coli* in surface waters in the Netherlands and Poland were detected in 11% [90] and 19% [91] of the samples, respectively. 

In our study, resistance to cefalexin (46%), ampicillin (40%), amoxicillin + clavulanic acid (42%), streptomycin (34%), and tetracycline (35%), were the highest (Figure 4). These antibiotics are considered clinically and agriculturally important, increasing the risk of complicated infections in swimmers, consumers of produce irrigated with contaminated waters, and livestock that might use these waters [85,92]. Resistance to carbapenems was low and only identified in three isolates from the North, specifically in Zgharta and Oyoun el Samak rivers (Figure 5). However, this should be considered a warning sign, because carbapenems are last-resort antibiotics for treating complicated life-threatening infections in humans [93]. Recently, multidrug-resistant *E. coli* that also harbored transmissible resistance to colistin, which is used to treat carbapenem-resistant *Enterobacteriaceae* (CRE) infections, was detected in irrigation water and sewage in the Beqaa region [30,37,39]. Taken together, it can be argued that continuous contamination might cause river water to become a reservoir for the evolution, emergence, and dissemination of MDR bacterial pathogens and ABR genetic determinants. 

## 5. Conclusions

To our knowledge, this study is the first nationwide assessment of fecal pollution and the dissemination of antibiotic-resistant *E. coli* in river water in Lebanon. The data show that most of the rivers in Lebanon are heavily contaminated by fecal indicator bacteria, which jeopardizes harnessing the full potential of these critical resources in irrigation and recreation. This is further confirmed by the detection of *E*. *coli* that were resistant to clinically and agriculturally important antibiotics. Although our study was cross-sectional and did not assess other factors like water flow, chemical contamination, and seasonal variation, the results indicate clearly that fecal pollution is severely impacting rivers in Lebanon. This study highlights the urgent need to implement proper wastewater management to preserve the safety and sustainability of river water in Lebanon. Our data also suggest that fecal pollution can be remediated because the majority of upstream locations were found to be less contaminated or acceptable. However, action must be taken immediately to prevent further deterioration of the rivers. Furthermore, there is a need to strengthen antimicrobial stewardship and enhance surveillance programs to study antibiotic resistance in environmental niches in Lebanon, which remains lacking. This issue is very important locally and regionally, because river water can also carry antibiotic-resistant bacteria across borders and into the Mediterranean basin. The assessment of the emergence and dissemination of antibiotic resistance in water and other environments in Lebanon would benefit greatly from future studies on the underlying genetic mechanisms of resistance. Finally, we call for adopting clear and strict guidelines and standards for water safety and to continuously monitor the quality of water in Lebanese rivers, which are essential contributors to public health and economy.

## Figures and Tables

**Table 1:** Sampling locations across the major rivers in Lebanon, upstream (U), midstream (M), and downstream (D). Sample identifiers (ID) are included for each sampling location. For example, Wak, Hed, and Ari represent Wadi Khaled, Hekr el Dahri, and Arida, which are upstream, midstream, and downstream of the Kabir river, respectively. Major rivers are listed in order from the North to the South of Lebanon. * Inaccessible: the location was across the Lebanese borders and could not be sampled.

Name of Major River	Sampling Site				
	Upstream (ID)	Midstream (ID)			Downstream (ID)
Kabir	Wadi Khaled (Wak)	Hekr el Dahri (Hed)			Arida (Ari)
Oustwein	Ain el Tineh (Aet)	Khuraybat al Jundi (Kaj)			Oustwein (Ous)
Bared	Fnaidek (Fna)	Oyoun el Samak (Oes)			Bared (Brd)
Abou Ali	Bcharri (Bch)	Zgharta (Zgh)			Abou Ali (Aba)
El Jawz	Tannourine (Tan)	Kaftoun (Kaf)			El Jawz (Jaw)
Assi	Ain el Zerqa (Aez)	Bejaj (Bej)			Labweh (Lab)
Ibrahim	Afqa (Afq)	Yahchouch (Yah)			Ibrahim (Ibr)
El Kalb	Faraya (Far)	Jeita (Jei)			El Kalb (Kal)
Beirut	Majdal Tarshish Aintoura (MTA)	Beirut (Bei)			Beirut Port (Bep)
Litani	Nabeh el Litani (Nal)	Bar Elias (Bae)	Jarmaq (Jar)	Mazraat Tamrah (Mat)	Qasmiye (Qas)
Damour	Nabaa al Safaa (Nas)	Jisr el Qadi (Jeq)			Damour (Dam)
Awali	Barouk (Bar)	Besri (Bes)			Awali (Awa)
Zahrani	Nabaa el Tasse (Net)	Habbouch (Hab)			Zahrani (Zah)
Hasbani	Hasbaya (Has)	Ibel al Saqi (Ias)		Wazzini (Waz)	Inaccessible *

## Data Availability

All the relevant data have been included in this study. We did not generate data that required public.
